# Ambulatory and stationary healthcare use in survivors of ARDS during the first year after discharge from ICU: findings from the DACAPO cohort

**DOI:** 10.1186/s13613-019-0544-5

**Published:** 2019-06-14

**Authors:** Susanne Brandstetter, Frank Dodoo-Schittko, Magdalena Brandl, Sebastian Blecha, Thomas Bein, Christian Apfelbacher, DACAPO study group, Johannes Bickenbach, Thorben Beeker, Tobias Schürholz, Jessica Pezechk, Jens Schloer, Ulrich Jaschinski, Ilse Kummer, Oliver Kuckein, Steffen Weber-Carstens, Anton Goldmann, Stefan Angermair, Krista Stoycheva, Jörg Brederlau, Nadja Rieckehr, Gabriele Schreiber, Henriette Haennicke, Friedhelm Bach, Immo Gummelt, Silke Haas, Catharina Middeke, Ina Vedder, Marion Klaproth, Michael Adamzik, Jan Karlik, Stefan Martini, Luisa Robitzky, Christian Putensen, Thomas Muders, Ute Lohmer, Rolf Dembinski, Petra Schäffner, Petra Wulff-Werner, Elke Landsiedel-Mechenbier, Daniela Nickoleit-Bitzenberger, Ann-Kathrin Silber, Maximilian Ragaller, Marcello Gama de Abreu, Alin Ulbricht, Linda Reisbach, Kai Zacharowski, Patrick Meybohm, Simone Lindau, Haitham Mutlak, Alexander Hötzel, Johannes Kalbhenn, Christoph Metz, Stefan Haschka, Stefan Rauch, Michael Quintel, Lars-Olav Harnisch, Sophie Baumann, Andrea Kernchen, Sigrun Friesecke, Sebastian Maletzki, Stefan Kluge, Olaf Boenisch, Daniel Frings, Birgit Füllekrug, Nils Jahn, Knut Kampe, Grit Ringeis, Brigitte Singer, Robin Wüstenberg, Jörg Ahrens, Heiner Ruschulte, Andre Gerdes, Matthias Groß, Olaf Wiesner, Aleksandra Bayat-Graw, Thorsten Brenner, Felix Schmitt, Anna Lipinski, Dietrich Henzler, Klaas Eickmeyer, Juliane Krebs, Iris Rodenberg, Heinrich Groesdonk, Kathrin Meiers, Karen Salm, Thomas Volk, Stefan Fischer, Basam Redwan, Martin Schmölz, Kathrin Schumann-Stoiber, Simone Eberl, Gunther Lenz, Thomas von Wernitz-Keibel, Monika Zackel, Frank Bloos, Petra Bloos, Anke Braune, Anja Haucke, Almut Noack, Steffi Kolanos, Heike Kuhnsch, Karina Knuhr-Kohlberg, Markus Gehling, Mathias Haller, Anne Sturm, Jannik Rossenbach, Dirk Schädler, Stefanie D’Aria, Christian Karagiannidis, Stephan Straßmann, Wolfram Windisch, Thorsten Annecke, Holger Herff, Michael Schütz, Sven Bercker, Hannah Reising, Mandy Dathe, Christian Schlegel, Katrin Lichy, Wolfgang Zink, Jana Kötteritzsch, Marc Bodenstein, Susanne Mauff, Peter Straub, Christof Strang, Florian Prätsch, Thomas Hachenberg, Thomas Kirschning, Thomas Friedrich, Dennis Mangold, Christian Arndt, Tilo Koch, Hendrik Haake, Katrin Offermanns, Patrick Friederich, Florian Bingold, Michael Irlbeck, Bernhard Zwissler, Ines Kaufmann, Ralph Bogdanski, Barbara Kapfer, Markus Heim, Günther Edenharter, Björn Ellger, Daniela Bause, Götz Gerresheim, Dorothea Muschner, Michael Christ, Arnim Geise, Martin Beiderlinden, Thorsten Heuter, Alexander Wipfel, Werner Kargl, Marion Harth, Christian Englmeier, Marius Zeder, Markus Stephan, Martin Glaser, Helene Häberle, Hendrik Bracht, Christian Heer, Theresa Mast, Markus Kredel, Ralf Müllenbach

**Affiliations:** 1 Medical Sociology, Institute for Epidemiology and Preventive Medicine, University of Regensburg, Dr.-Gessler-Str. 17, 93051 Regensburg, Germany; 2 Department of Anesthesia and Operative Intensive Care, University Hospital Regensburg, Franz-Josef-Strauss-Allee 11, 93053 Regensburg, Germany; 3 Institute of Social Medicine and Health Economics (ISMHE), University of Magdeburg, Leipziger Str. 44, 39120 Magdeburg, Germany

**Keywords:** Healthcare use, Ambulatory health care, Stationary health care, Health services research, ARDS, Critical illness, Post-ICU

## Abstract

**Background:**

For many survivors of acute respiratory distress syndrome (ARDS), the process from discharge from intensive care unit (ICU) to recovery is long and difficult. However, healthcare use after discharge from ICU has received only little attention by research. This study sets out to investigate the extent of ambulatory and stationary healthcare use among survivors of ARDS in Germany (multicenter DACAPO cohort) and to analyze predictors of stationary healthcare use.

**Results:**

A total of 396 survivors of ARDS provided data at 1 year after discharge from ICU. Fifty percent of 1-year survivors were hospitalized for 48 days or longer after discharge from ICU, with 10% spending more than six out of 12 months in stationary care. The duration of hospitalization increased significantly by the length of the initial ICU stay. All participants reported at least one outpatient visit (including visits to general practitioners), and 50% contacted four or more different medical specialties within the first year after discharge from ICU.

**Conclusions:**

For most of the patients, the first year after ARDS is characterized by an extensive amount of healthcare utilization, especially with regard to stationary health care. These findings shed light on the substantial morbidity of patients after ARDS and contribute to a better understanding of the situation of patients following discharge from ICU.

## Background

Acute respiratory distress syndrome (ARDS) is a severe life-threatening condition which requires intensive care treatment and in the majority of patients mechanical ventilation. Hospital mortality varies—depending on the severity of ARDS—between 35 and 46% [1]. For some survivors of ARDS, the process to full recovery and return-to-work is long and difficult: Following discharge from the intensive care unit (ICU), limitations in functioning and health-related quality of life are common [2, 3] and many patients suffer from psychological sequelae [4, 5]. Studies have also shown that impairments can persist over years [6, 7].

Against the background of long-term morbidity after ARDS, a better knowledge of healthcare use among survivors of ARDS seems crucial. Healthcare use represents a multilayered construct: It is not only a reflection of patients’ individual characteristics, such as health status and perceived need of treatment, but also of characteristics of the healthcare system, e.g., the availability of and the access to specific services [8]. Information on healthcare use contributes to the estimation of healthcare expenditures and can be useful for revealing situations of regional practice variation as well as under- or over-supply [9]. From a health services research perspective, healthcare use is also a relevant outcome in itself.

Only few previous studies from Canada and the USA have investigated healthcare use in survivors of ARDS [10–12]. Between 1998 and 2001, a Canadian cohort study included 109 ARDS survivors, 39% of which had been readmitted to hospital during the first 2 years following ARDS [10]. Two more recent cohort studies from the USA report data for the first year after discharge from ICU: 40% out of 839 [11] and 52% out of 138 ARDS survivors [12] reported at least any hospitalization, respectively.

It has to be noted, though, that all these cohort studies applied—in part strict—exclusion criteria, such as the diagnosis of psychiatric or neurological disorders or low predicted life expectancy due to comorbidity, and possibly underestimated the account of healthcare utilization following ARDS. In addition, it is well known that the organization of healthcare systems can affect healthcare use making it difficult to compare findings between studies from different countries.

Healthcare use after ARDS in Germany has not been investigated which is why we set out to address this research gap by (1) describing the extent to which survivors of ARDS use ambulatory and stationary healthcare services during the first year after discharge from ICU and by (2) analyzing socio-demographic and disease-related predictors of stationary healthcare utilization.

## Methods

### Study design

This study analyzes data on healthcare utilization among patients after ARDS from the DACAPO study (“Surviving ARDS: the influence of quality of care and individual patient characteristics on health-related quality of life”), a multicenter patient cohort study whose primary aim was to investigate the influence of quality of care on health-related quality of life and return-to-work in survivors of ARDS. The study procedures, baseline characteristics and profile of the cohort are described in more detail elsewhere [13–15]. Briefly, patients with ARDS were included in the study during their stay in the ICU of a participating clinic in Germany. After discharge from ICU, patients were recontacted at 3, 6 and 12 months and asked to complete comprehensive self-report questionnaires.

The study was approved by the Ethics Committee of the University of Regensburg (file number: 13-101-0262) and (if required) additionally by the Ethics Committees of the participating hospitals.

### Sample

Between September 2014 and April 2016, 1225 patients with ARDS from 61 hospitals all over Germany were included in the study. Inclusion criteria were the presence of ARDS (according to the criteria of the Berlin definition [16]) and being at least 18 years old. In order to ensure generalizability of the results, no exclusion criteria were applied. Patients or their caregivers/legal guardians were approached during the ICU stay and asked to provide written informed consent. In cases where caregivers/legal guardians consented to the participation in the study, patients had to confirm this preliminary consent after discharge from ICU.

**Fig. 1:**
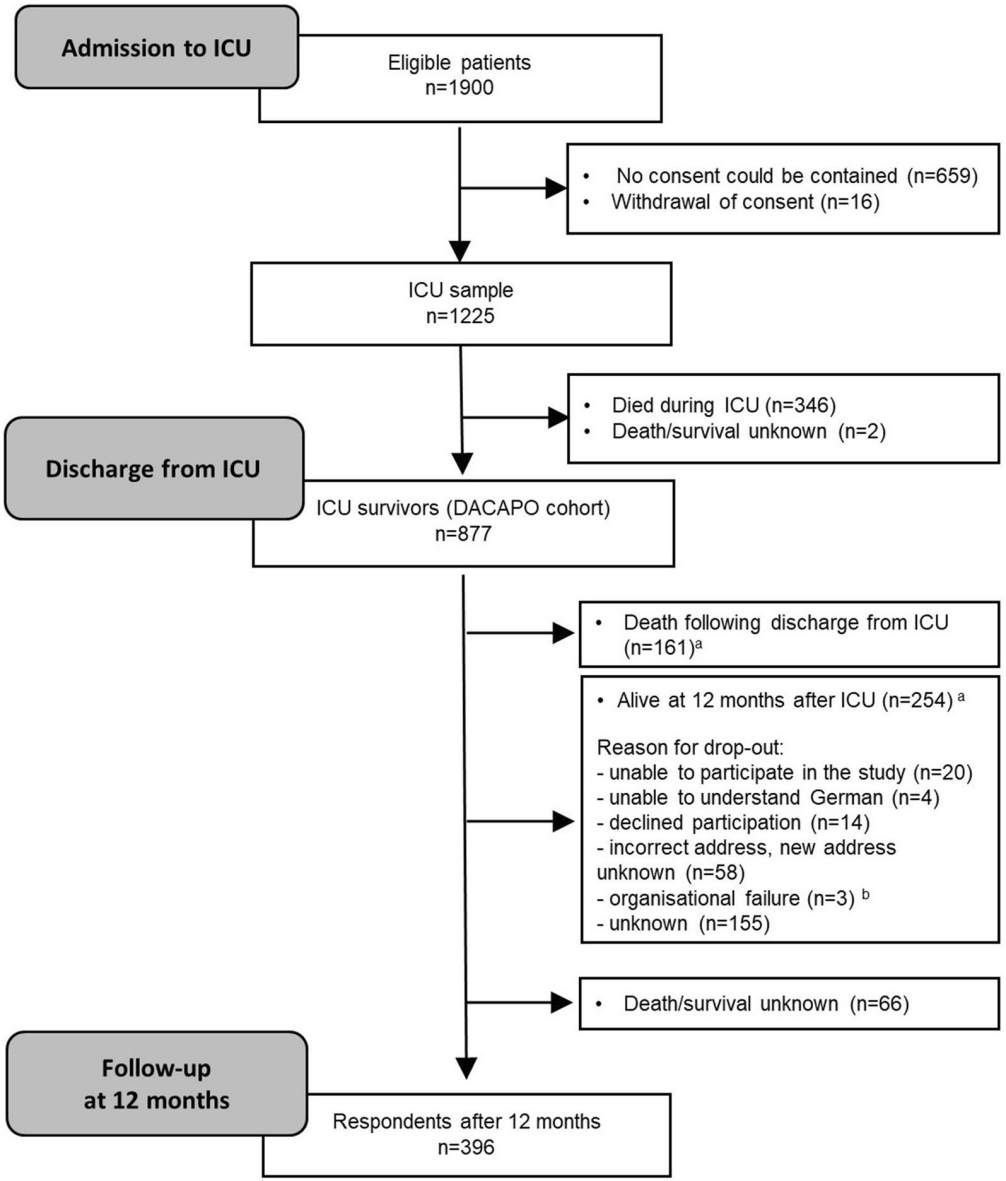
Patient flow. *Notes*: *ICU* intensive care unit. ^a^For all patients who were lost to follow-up, survival was assessed via local municipal population registries. ^b^Written informed consent and patient data were transferred to the study centre with a delay of more than 12 months; thus, follow-up measurement was not possible within the scheduled follow-up period

Out of 877 ICU survivors, 396 (45%) returned the questionnaire at 1 year after discharge from ICU. Figure 1 depicts the patient flow over the course of the study and gives an overview of the sample size at different time points. The most frequent reason for study drop out was death during the period after discharge from ICU (*N* = 161). Other reasons included the inability to complete the questionnaire (insufficient knowledge of German, incapable due to morbidity), the lack of a person who could provide proxy reports, withdrawal of consent or invalid addresses.

### Data sources and data collection

This study uses data from two data sources:

I. Patients’ sociodemographic, disease and treatment-related characteristics as well as information on referral to and discharge from ICU were reported by study physicians/nurses from the individual ICUs using the electronic data entry system OpenClinica (OpenClinica, LLC; https://www.openclinica.com/).
II. Information on healthcare utilization (comprising inpatient stays following ICU discharge and outpatient physician visits) was assessed by self-report questionnaires at 6 months and at 1 year after discharge from ICU.

Plausibility checks of self-report data were performed and comprised the following procedures: Data which was reported both by study participants and by study personnel from the participating ICUs were compared, in case of non-concordance information provided by study personnel was considered valid. If participants completed questionnaires both at 6 months and at 1 year, information of these two questionnaires was compared with each other. In cases of non-concordance, the information which was provided earlier was considered valid. Further, it was checked whether time spans of inpatient stays were overlapping or whether the reported events followed the expected order (ICU, inclusion in the study, referral from ICU, etc.). Implausible information was handled as missing values. If the duration of single inpatients stays was implausible, the overall duration of inpatient stays was not calculated.

### Measures

I. Sociodemographic, disease and treatment-related characteristics

Sociodemographic data comprise age, sex, education, living situation, employment situation before ICU and health insurance. Disease- and treatment-related characteristics include cause of ARDS (pulmonary, extrapulmonary, other), severity of ARDS (mild, moderate, severe [16]), diagnosis of ARDS (in a participating hospital, in a transferring hospital), scores indicating disease severity and morbidity at admission to ICU (SOFA [17], SAPS-II [18]), length of stay in the hospital and the ICU (days) and mechanical ventilation at discharge from ICU.

II. Healthcare use

The German healthcare system is mainly separated into three broad sectors: ambulatory medical treatment carried out by hospitals or by physicians in private practice, inpatient treatment provided by hospitals and rehabilitative treatment provided by rehabilitation facilities. This separation goes along with differences in underlying legislation and funding agencies [19]. Albeit the healthcare sectors have different tasks and pursue different objectives, there is also overlap in the services provided and for someone utilizing a certain healthcare service this differentiation might not be obvious. As we use self-report data of healthcare use for the purpose of our analyses, we refer to the following categorization of healthcare use.

*Inpatient stays/stationary care* Patients were asked to provide information on all inpatient stays following discharge from ICU in chronological order. Dates of the stays as well as name and place of the institution were assessed. This information was used to calculate the number of inpatient stays and the sum of days patients spent in stationary care. A hospital stay was considered an inpatient stay if the patient spent at least one night in the hospital. Stays in rehabilitation units were also considered inpatient stays.

*Outpatient physician visits/ambulatory care* Patients were asked to report whether and how often they had visited primary care and specialized physicians since discharge from ICU. This included both physicians in private practice and hospitals offering ambulatory care. The specialty types considered in the questionnaire comprised general practitioners, internists, obstetricians/gynecologists, ophthalmologists, orthopedists, otolaryngologists, neurologists/psychiatrists, psychotherapists, surgeons, dermatologists, radiologists, dentists and a category for “other specialty.” Data were analyzed regarding the number of different specialties which have been contacted and to the total number of outpatient visits, separately for all visits and for all visits excluding general practitioner visits.

### Statistics

Descriptive statistics were computed. Patients’ characteristics are presented as frequencies and percentages for categorical or medians and interquartile ranges for continuous variables, respectively. Data on duration or frequency of health service utilization are provided as median and interquartile ranges.

This study describes healthcare utilization by different parameters. Duration of hospitalization was considered the most important outcome given the amount of healthcare costs and the severity of limitations for the patient’s life associated with hospitalization. Accordingly, the analysis of determinants of healthcare utilization was restricted to this outcome. In order to account for the extreme overdispersion of count data on duration of hospitalization, a multivariable negative binomial regression model was computed for analyzing the association between sociodemographic and disease-related variables with duration of hospitalization. We applied a two-step approach for the selection of independent variables: First, socio-demographic and disease-related variables were selected. Second, these variables were tested using an empirical criterion. All variables which were significantly associated (*p *< 0.05) with the outcome in univariable models were included in the final multivariable model. Incidence rate ratios (IRRs) and 95% confidence intervals (CIs) are provided.

All analyses were computed using Stata 14.1.

## Results

### Patient characteristics

Sociodemographic as well as disease- and treatment-related characteristics of study participants who returned the self-report questionnaire at 1 year after discharge from ICU are displayed in Table 1. Two-thirds of persons were male. Median age at admission to ICU was 56 years (IQR 47–65). The vast majority had a moderate (46%) or severe form (43%) of ARDS (according to the classification provided by the Berlin definition [16]).

**Table 1:** Sociodemographic and disease-related characteristics of study participants (*N* = 396 respondents at 1-year follow-up)

	*N*	
Sex male, *N* (%)	396	264 (66.7)
Age (years), (Md, IQR)	396	56 (47–65)
Educational level	337	
No school leaving certificate, *N* (%)		7 (2.1)
Not yet a school leaving certificate, *N* (%)		2 (0.6)
Secondary school leaving certificate, *N* (%)		131 (38.9)
Intermediate school leaving certificate, *N* (%)		121 (35.9)
University entrance level, *N* (%)		76 (22.6)
Education score^a^: (Md, IQR)	354	3.6 (3.0–3.6)
Employment situation before onset of ARDS	347	
Full time, *N *(%)		152 (43.8)
Part time,* N* (%)		31 (8.9)
Irregular, *N* (%)		5 (1.4)
Not employed/retired, *N* (%)		159 (45.8)
Nationality	382	
German, *N* (%)		368 (96.3)
Other, *N* (%)		14 (3.7)
Living with a partner *N* (%)	380	294 (77.4)
Health insurance	363	
Statutory, *N* (%)		316 (87.0)
Private, *N* (%)		42 (11.6)
Other, *N* (%)		5 (1.4)
SAPS-II score at admission (without GCS), Md (IQR)	361	38 (31–47)
SOFA score at admission (without GCS), Md (IQR)	345	8 (6–10)
Cause of ARDS	374	
Pulmonary, *N* (%)		320 (85.6)
Extrapulmonary, *N* (%)		54 (14.4)
Diagnosis of ARDS	386	
Diagnosis in participating ICU, *N* (%)		232 (60.1)
Diagnosis in other ICU (transferred after diagnosis to participating ICU), *N* (%)		154 (39.9)
Severity of ARDS	387	
Mild,* N* (%)		39 (10.1)
Moderate, *N* (%)		180 (46.5)
Severe, *N* (%)		168 (43.4)
Length of ICU stay until discharge (days), (MD, IQR)	379	23 (14–36)
Length of hospital stay until discharge (days), (MD, IQR)	367	27 (17–40)
Mechanical ventilation at discharge from ICU, *N* (%)	387	52 (13.4)

*Md* median, *IQR* interquartile range, *ARDS* acute respiratory distress syndrome, *ICU* intensive care unit, *SAPS-II* Simplified Acute Physiology Score-II, *SOFA* sequential organ failure assessment, *GCS* Glasgow Coma Scale

^a^Derived from educational and professional levels [36]

### Descriptive results

*Discharge from ICU* When discharged from ICU, most patients (59%) were referred within the same hospital, 41% to another hospital or to a rehabilitation unit. Only one person was discharged home.

**Fig. 2:**
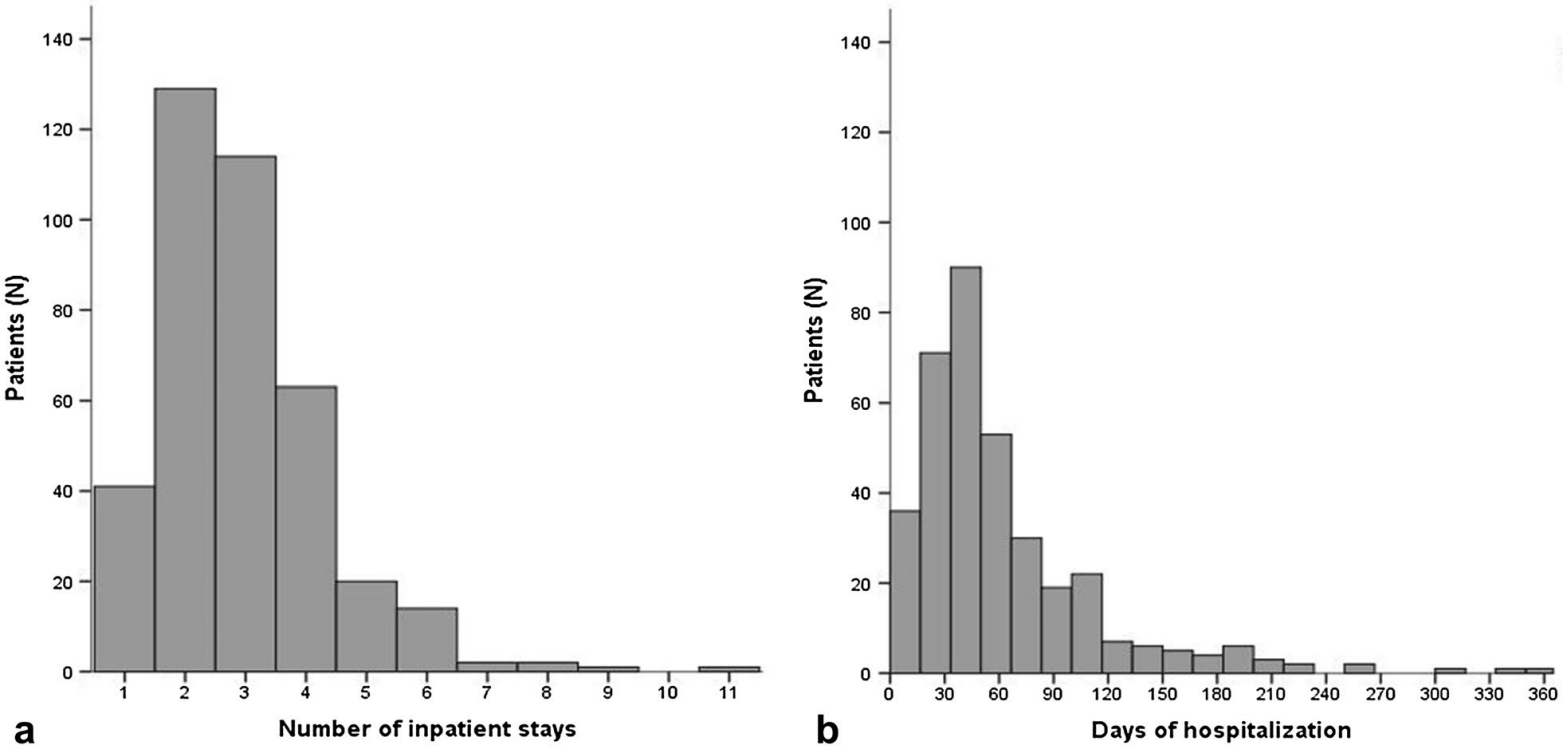
Number of inpatient stays (**a**) and days of hospitalization (**b**) during the first year after discharge from ICU. *Notes*: *N* = 387 for inpatient stays, *N* = 359 for days of hospitalization; inpatient stays included stays in hospitals (ICU or normal ward) and rehabilitation units; subsequent stays were considered distinct from each other if there was a change in the hospital or if a stay within a hospital was interrupted by at least one night at home. Referrals within one institution (e.g., from normal ward to ICU and vv.) were considered a single stay

*Inpatient stays* The number of patients’ individual hospital stays is depicted in Fig. 2a. Including the initial hospital stay for the treatment of ARDS, the median number of inpatient stays within 1 year after discharge from ICU was 3 (IQR 2–4). These stays comprised re-admissions to both ICUs and normal wards, admissions for medical problems not related to the initial ICU stay and rehabilitative measures. Only 10% of patients had no additional hospital stay after discharge from the clinic where they had been treated for ARDS.

Within the first year after discharge from ICU, the median number of days of hospitalization was 48 (IQR 31–76) (see Fig. 2b). The variability was high: Nearly 10% of patients were hospitalized for a period longer than 6 months.

*Outpatient visits* All study participants reported at least one outpatient visit to a general practitioner or any other physician during the first year after discharge from ICU. Most participants contacted physicians from various specialties: The median number of different medical specialties visited was 4 (IQR 3–6) including general practitioners and 3 (IQR 2–5) without general practitioners.

Table 2 provides an overview on which percentage of study participants utilized the different medical specialties. The most frequently contacted medical specialties were general practitioners with 93% of study participants reporting a visit, followed by internists with 56%. 37% of study participants had at least one visit to a neurologist, psychiatrist or psychotherapist.

**Table 2:** Study participants’ outpatient visits during the first year after discharge from ICU according to medical specialty

	% of participants reporting at least one visit
General practitioner	93.5
Internist	56.5
Obstetrician/gynecologist	41.1^a^
Ophthalmologist	32.6
Orthopedist	21.8
Otolaryngologist	25.4
Neurologist, psychiatrist	31.6
Psychotherapist	14.0
Surgeon	20.7
Dermatologist	17.9
Radiologist	36.8
Dentist, orthodontist	58.0
Other specialty^b^	13.2
Any specialty	100.0

Multiple answers possible

100% (*N* = 386) refers to all participants who provided any information about outpatient visits

^a^Analyzed only for women

^b^Most frequently reported other specialities: urologist, oncologist

Overall, the median number of outpatient visits during the first year after discharge from ICU was 15, with a high variability between participants (IQR 8–25). The median number of outpatient visits to a general practitioner was 7 (IQR 4–12), and the median number for visits to physicians from any other specialty was 8 (IQR 4–14).

### Analytical results

In univariable analyses, indicators of disease severity (SOFA score, SAPS-II score), transferral from another hospital to the study hospital, length of ICU stay, overall length of hospital stay until discharge from ICU (including ICU stay) and mechanical ventilation at discharge from ICU were significantly associated with the number of days hospitalized during the first year after ICU (Table 3). There was no significant association with any of the sociodemographic variables.

**Table 3:** Univariable negative binomial regression analyses of days of hospitalization after discharge from ICU

	IRR	SE	95% CI	*p*
Sex male	1.026	0.087	0.87–1.21	0.765
Age at admission to ICU (years)	1.002	0.003	0.99–1.01	0.527
Education score^a^	1.049	0.034	0.98–1.12	0.143
Employment situation before onset of ARDS:				
Full time	Reference			
Part time	0.855	0.137	0.62–1.17	0.327
Irregular	0.832	0.326	0.38–1.79	0.639
Not employed/retired	1.096	0.100	0.91–1.31	0.317
Health insurance				
Statutory	Reference			
Private	1.089	0.138	0.85–1.40	0.498
Other	0.575	0.219	0.27–1.21	0.147
Living with a partner	1.115	0.110	0.92–1.35	0.273
Nationality: German	1.371	0.310	0.88–2.14	0.164
Transferred from other ICU	1.205	0.100	1.02–1.42	0.024
Severity of ARDS:				
Mild	Reference			
Moderate	0.874	0.123	0.66–1.15	0.339
Severe	0.860	0.122	0.65–1.13	0.287
Cause of ARDS: extrapulmonary	1.259	0.153	0.99–1.60	0.059
SAPS-II at admission to ICU (without GCS)	1.007	0.004	1.00–1.01	0.047
SOFA score at admission to ICU (without GCS)	1.026	0.012	1.00–1.05	0.029
Length of ICU stay (10 days)^b^	1.112	0.024	1.06–1.16	< 0.001
Length of hospital stay (10 days)^b^	1.113	0.022	1.07–1.16	< 0.001
Mechanical ventilation at discharge	1.376	0.168	1.08–1.75	0.009

*IRR* incidence rate ratio, *SE* standard error, *95% CI* 95% confidence interval, *SAPS-II* Simplified Acute Physiology Score-II, *SOFA* sequential organ failure assessment, *GCS* Glasgow Coma Scale

^a^Derived from educational and professional levels [36]

^b^Including stay in transferring hospital

Table 4 presents the results of the multivariable analysis. Since the predictor variables “length of hospital stay” and “length of ICU stay” were highly correlated (*r* = 0.9), only length of ICU stay was included in the multivariable model. Duration of hospitalization after discharge from ICU was significantly associated with length of ICU stay (incidence rate ratio (IRR): 1.10, 95% CI 1.05–1.15), with each 10 days of ICU stay prolonging the duration of hospitalization after discharge from ICU by 10%. All other associations were attenuated in the multivariable model and did not reach statistical significance.

**Table 4:** Multivariable negative binomial regression analysis of days of hospitalization after discharge from ICU

	IRR	SE	95% CI	*p*
Transferred from other ICU	1.160	0.100	0.98–1.37	0.083
SAPS-II at admission to ICU (without GCS)	1.002	0.004	0.99–1.01	0.587
SOFA score at admission to ICU (without GCS)	0.997	0.014	0.97–1.02	0.833
Length of ICU stay (10 days)^a^	1.098	0.025	1.05–1.15	< 0.001
Mechanical ventilation at discharge	1.178	0.143	0.97–1.49	0.179

*IRR* incidence rate ratio, *SE* standard error, *95% CI* 95% confidence interval, *ICU* intensive care unit, *SAPS-II* Simplified Acute Physiology Score-II, *SOFA* sequential organ failure assessment, *GCS* Glasgow Coma Scale

^a^Including stay in transferring hospital

## Discussion

Until now, healthcare utilization following ARDS has received only little attention by research and healthcare providers. Our study contributes to a better understanding of the situation of patients after ARDS by providing a comprehensive description of both stationary and ambulatory healthcare use during the first 12 months after discharge from ICU in a large German cohort of 1-year survivors of ARDS: We found that 50% of 1-year survivors were hospitalized for 48 days or longer after discharge from ICU. Ten percent spent even more than six out of 12 months in stationary care. The duration of hospitalization increased significantly by the length of the initial ICU stay. Remarkably, none of the other investigated variables were associated with the duration of hospitalization. Study participants reported also a substantial amount of outpatient physician visits, with 50% of former ARDS patients having contact to four or more different medical specialties (including general practitioners) within a 1-year period.

### The extent of healthcare utilization in 1-year survivors of ARDS

Findings on healthcare utilization have to be discussed in view of the respective healthcare system as comparability across countries is severely impeded due to structural differences between systems [19]: The German healthcare system is characterized by a separation between the hospital and the outpatient sector as well as between acute care and rehabilitative treatment. The number of hospital beds per inhabitant is larger than that in most of the other European countries [20] and also the duration of hospital stays is longer [21]. Health insurance is mandatory, and the access to and the reimbursement of services are comprehensive [19]. With regard to patients after ARDS, their situation is characterized by the following specific circumstances in Germany: Albeit the rehabilitation system is elaborated [22], there are no follow-up clinics or rehabilitation units that are specialized in the care of former ARDS patients. Thus, the choice of a clinic or a rehabilitation unit is informed by the underlying disease which has caused ARDS or it follows practical considerations such as whether an institution is able to deal with a patient’s health status and need of care as well as the currently available capacities.

Acknowledging these specifics of the German healthcare system which are related to the extent of healthcare utilization, we refer to a sample of sepsis survivors and to a representative sample from the general population in Germany which might help to interpret the data on healthcare use of patients after ARDS:

The SMOOTH study included 291 survivors of sepsis and investigated the effects of a primary-care-based intervention [23]. A variety of secondary outcomes were assessed, among others measures of healthcare utilization. With respect to stationary healthcare utilization, participants from that study had values far below these of our study among ARDS survivors. During the first 6 months after discharge from ICU, former sepsis patients from the control group (care as usual) spent a median time of 8 days (IQR 0–32) in a hospital and of 0 days (IQR 0–21) in a rehabilitation clinic. During the months seven to 12 after discharge, the median number of days of both hospital and rehabilitation stay was 0 [24]. However, the number of outpatient visits was comparable to our study.

The representative German Health Interview and Examination Survey for Adults (DEGS1) found that 16% of the general population was hospitalized at least once during a 1-year period for on average 9.7 nights. Not unexpectedly, this is in stark contrast to the findings from our sample: 90% of participants had one or more additional hospital stays (including rehabilitation) within the first year after discharge from ICU. With respect to healthcare use in the outpatient sector, the DEGS1 survey found that the mean number of outpatient visits (including visits to general practitioners) per year was 9.2 in the general population. However, persons over the age of 70 years and people with poor self-rated health had a mean number of 11.5 and 15.0 visits, respectively [25]. The latter corresponds to the finding obtained from our cohort and reflects a substantial morbidity among survivors of ARDS.

### Concurrent treatment by different healthcare providers

The majority of patients in our sample had several inpatient stays and visited also physicians from a variety of disciplines. The number of different contacted medical specialist groups is elevated in our cohort as compared to the general population [25]. This finding may reflect many comorbidities or ARDS sequelae which compromise different organ systems and impair patients’ functioning at various levels. For the latter, in the last decade, the term post-intensive care syndrome (PICS) has been proposed [26]. PICS summarizes new or worsening impairments in physical, mental and cognitive functioning which can occur after prolonged treatments in the ICU and are often not sufficiently covered by healthcare.

But the relatively high number of different contacted medical specialties may also be read as frequent referrals between specialties pointing out the need for other or additional treatments tailored to the needs of former critically ill patients. Whichever way, concurrent treatment provided by different physicians is likely to make the flow of information more difficult and is a challenge for both the involved healthcare providers and the patients.

### Utilization of mental health care

The percentage of patients who visited a neurologist/psychiatrist or psychotherapist is of special interest. The still-existing stigma associated with mental illness [27], low rates of help seeking for mental health problems [28] and concerns regarding the availability of mental health care [29] suggest that patients’ access to these specialties might be more difficult as compared to others. However, the percentages of 32% and 14% in our study are quite high as compared to 8% and 4% of people from the general population in Germany who had contact to a psychiatrist/neurologist or psychotherapist within a 1-year period, respectively [25]. As help seeking for mental disorders can be difficult for some people, these high percentages of patients after ARDS who utilized mental health care are likely to reflect a special need of this population. A systematic review found that mental disorders are common in people after ARDS: The prevalences for depression, anxiety and post-traumatic stress disorder (PTSD) approximately range between 20 and 40% [4]. A more recent study reported that even two-thirds of patients after ARDS are experiencing symptoms of mental disease [30].

## Strengths and limitations

To the best of our knowledge, this is the first study on healthcare utilization in survivors of ARDS in Germany. It used primary data and investigated patients’ healthcare utilization both in the inpatient and the outpatient sectors.

In contrast to other cohorts of ARDS survivors, no exclusion criteria (e.g., with regard to comorbidity or to estimated life expectancy) were applied. Our study sample corresponded to the characteristics expected for ARDS cohorts with regard to the distribution of sex and age [15]. However, the liberal inclusion criteria might have led to a higher proportion of severely ill patients—as can be seen by 40% of persons in our study sample which had a severe form of ARDS—and to high extents of healthcare utilization following the ICU stay.

Information on healthcare utilization after discharge from ICU was gathered through self-report questionnaires. Extensive plausibility checks were conducted, and the majority of patients were found to provide apparently comprehensive and detailed accounts on their contacts with healthcare providers. Nevertheless, we cannot exclude that data on healthcare utilization are incomplete or imprecise and by using self-report data on inpatient stays we were not able to differentiate between different types of hospitals and rehabilitation units. With regard to the findings obtained in our study, this limitation of self-report data could have led to an underestimation of the extent of healthcare utilization. It seems unlikely that persons reported non-existent hospital stays or contacts to a physician; but contacts with the healthcare system might have been omitted or not been correctly recalled—particularly with regard to ambulatory health care and in people who used healthcare services extensively [31, 32].

Our study focused on two major aspects of healthcare use (inpatient stays and ambulatory visits); however, the use of other health services (such as medication, medical aids and remedies, nursing care, etc.) was not considered.

In addition, we were not able to depict the mutual referrals of patients (e.g., between stationary and outpatient care or between different specialties), whether and how the various contacts with the healthcare system were interrelated and which were the reasons for the use of the various services. Our study does not allow for the differentiation between healthcare uses due to sequelae of ARDS or the ICU stay and due to any other complaints.

It should be noted that the sample for this study was people who survived the first year after ARDS and responded to the questionnaire. Thus, our study gives important insights into the health and living situation of long-term survivors of ARDS, but conclusions about healthcare utilization caused by ARDS cannot be drawn as we lack information on persons who have died during the first year after ARDS. However, one might speculate that these persons utilized health services even more often. Further, a considerable proportion of study participants were lost to follow-up. Unfortunately, loss to follow-up is a problem in many studies investigating long-term survivors [e.g., 33–35], and compromises the external validity of our findings.

In terms of clinical implications, clinicians should be aware that longer ICU stays entail the need to utilize many further health services in patients who survive ARDS. Future studies should additionally supplement self-report data with routine data provided by administrations or health insurances. This would allow for a more detailed description of healthcare use (e.g., the type and specialization of an institution) and an assessment of underlying reasons (e.g., main diagnosis).

## Conclusion

For many patients, the first year after ARDS is characterized by an extensive amount of healthcare utilization, especially with regard to stationary health care. The length of the initial ICU stay was associated with the duration of hospitalization during the first year after ARDS.

## Data Availability

Data are available upon request from the original data holders (the two principal investigators (PIs) TB and CA).

## Abbreviations

ARDS: acute respiratory distress syndrome
CI: confidence interval
ICU: intensive care unit
M: mean
IQR: interquartile range
IRR: incidence rate ratio
SD: standard deviation
